# Fractal analysis of 4D dynamic myocardial stress-CT perfusion imaging differentiates micro- and macrovascular ischemia in a multi-center proof-of-concept study

**DOI:** 10.1038/s41598-022-09144-6

**Published:** 2022-03-24

**Authors:** Florian Michallek, Satoshi Nakamura, Hideki Ota, Ryo Ogawa, Takehito Shizuka, Hitoshi Nakashima, Yi-Ning Wang, Tatsuro Ito, Hajime Sakuma, Marc Dewey, Kakuya Kitagawa

**Affiliations:** 1 Department of Radiology, Charité - Universitätsmedizin Berlin, Corporate Member of Freie Universität Berlin, Humboldt-Universität zu Berlin, and Berlin Institute of Health, Charitéplatz 1, 10117 Berlin, Germany; 2 Department of Radiology, Mie University Graduate School of Medicine, Mie, Japan; 3 Department of Advanced MRI Collaborative Research, Tohoku University Graduate School of Medicine, Miyagi, Japan; 4 Saiseikai Matsuyama Hospital, Matsuyama, Japan; 5 Takasaki General Medical Center, Takasaki, Japan; 6 National Hospital Organization Kagoshima Medical Center, Kagoshima, Japan; 7 Peking Union Medical College Hospital, Beijing, China; 8 Kobe University Graduate School of Medicine, Kobe, Japan; 9 DZHK (German Centre for Cardiovascular Research), partner site Berlin, Berlin, Germany; 10 Department of Advanced Diagnostic Imaging, Mie University Graduate School of Medicine, Mie, Japan

**Keywords:** Diagnostic markers, Ischaemia, Image processing

## Abstract

Fractal analysis of dynamic, four-dimensional computed tomography myocardial perfusion (4D-CTP) imaging might have potential for noninvasive differentiation of microvascular ischemia and macrovascular coronary artery disease (CAD) using fractal dimension (FD) as quantitative parameter for perfusion complexity. This multi-center proof-of-concept study included 30 rigorously characterized patients from the AMPLIFiED trial with nonoverlapping and confirmed microvascular ischemia (n_micro_ = 10), macrovascular CAD (n_macro_ = 10), or normal myocardial perfusion (n_normal_ = 10) with invasive coronary angiography and fractional flow reserve (FFR) measurements as reference standard. Perfusion complexity was comparatively high in normal perfusion (FD_normal_ = 4.49, interquartile range [IQR]:4.46–4.53), moderately reduced in microvascular ischemia (FD_micro_ = 4.37, IQR:4.36–4.37), and strongly reduced in macrovascular CAD (FD_macro_ = 4.26, IQR:4.24–4.27), which allowed to differentiate both ischemia types, p < 0.001. Fractal analysis agreed excellently with perfusion state (κ = 0.96, AUC = 0.98), whereas myocardial blood flow (MBF) showed moderate agreement (κ = 0.77, AUC = 0.78). For detecting CAD patients, fractal analysis outperformed MBF estimation with sensitivity and specificity of 100% and 85% versus 100% and 25%, p = 0.02. In conclusion, fractal analysis of 4D-CTP allows to differentiate microvascular from macrovascular ischemia and improves detection of hemodynamically significant CAD in comparison to MBF estimation.

## Introduction

Chronic myocardial ischemia has been recognized as a disease complex with different underlying pathophysiological entities involving the full range of vascular scales from the larger vessels (i.e., macrovascular coronary artery disease, CAD) to microcirculation. In microvascular ischemia, different concurring mechanisms have been observed including microvascular atherosclerosis and primary microvascular dysfunction (CMD)^1–3^. Microvascular ischemia is, most commonly, the result of diffuse CAD involving microvessels and can be detected as “subendocardial ischemia” by noninvasive perfusion imaging^4^. In less frequent primary microvascular dysfunction, the inability of the small arterial vessels and arterioles to regulate tissue perfusion in response to stimuli associated with exercise or external pharmacological induction typically results in homogenously reduced stress perfusion with a diffuse perfusion gradient^5,6^. Importantly, microvascular impairment affects a large proportion of patients with stable angina and is associated with a significantly higher rate of major adverse clinical events, especially in women^7,8^. Noninvasive clinical workup comprises both anatomical and physiological testing using CT angiography, SPECT, PET, echocardiography, MRI, and their combinations, e.g., PET-MRI^9^. Moreover, four-dimensional dynamic CT perfusion imaging (4D-CTP) has been introduced to assess functional relevance of anatomical stenoses to the myocardium^10^. However, noninvasive identification of a microvascular component in chronic myocardial ischemia is still challenging in clinical routine but would be beneficial in guiding clinical management and estimating individual prognosis^11^.

Many biological structures can be considered fractals due to their self-similar and scale-invariant organization, e.g., vascular trees^12,13^, cardiac dynamics^14,15^, and perfusion as a physiological process^16,17^. Geometrical complexity – or chaos – found in physiological myocardial perfusion is a well-recognized phenomenon and can be described using fractal geometry^12,16,17^. Animal studies using radioactively labelled microspheres provided insights into the self-similar and scale-invariant properties of myocardial perfusion, which form the basis for fractal analysis (FA)^16^. This concept has been transferred to radiological imaging^18^ and FA has shown potential to differentiate between macro- and microvascular ischemia on cardiac MRI^4,19,20^. The organization of vascular structure, both anatomically and functionally, comprises a number of different scales, including large to small arteries, pre-arterioles and arterioles, precapillary segments, the capillary bed and the venous drainage^21^. FA is suitable to characterize such multiscale structures based on fractal dimension (FD), which can be interpreted as a quantitative measure of geometrical complexity or chaos, in this case for characterizing perfusion patterns^12,16^. Therefore, FA might be suitable to identify the vascular scales impaired in individual patients, thereby differentiating between macrovascular ischemia (i.e., CAD) and microvascular ischemia (i.e., CMD) in patients with chronic myocardial ischemia.

This study explains how fractal principles of perfusion using FA of 4D-CTP might differentiate ischemia pathophysiology in rigorously characterized, nonoverlapping groups of patients with normal perfusion, microvascular CMD and macrovascular CAD with an invasive standard of reference.

## Methods

### Patients

Data from the prospective AMPLIFiED multicenter study (registered identification number UMIN000016353) were analyzed. Briefly, AMPLIFiED is a prospective, multicenter study including a total of 174 patients with suspected or confirmed, stable coronary artery disease and a clinical indication for invasive coronary angiography and was conducted to assess the diagnostic performance of four-dimensional stress dynamic CT perfusion imaging (4D-CTP). In this pilot study, we investigated patients with accurately characterized and clinically confirmed ischemia types to establish reference values for FD. Our cohort consisted of 30 patients with microvascular ischemia (n = 10), macrovascular ischemia (n = 10), and normal perfusion (n = 10).

### Definition of patient groups

**Figure 1:**
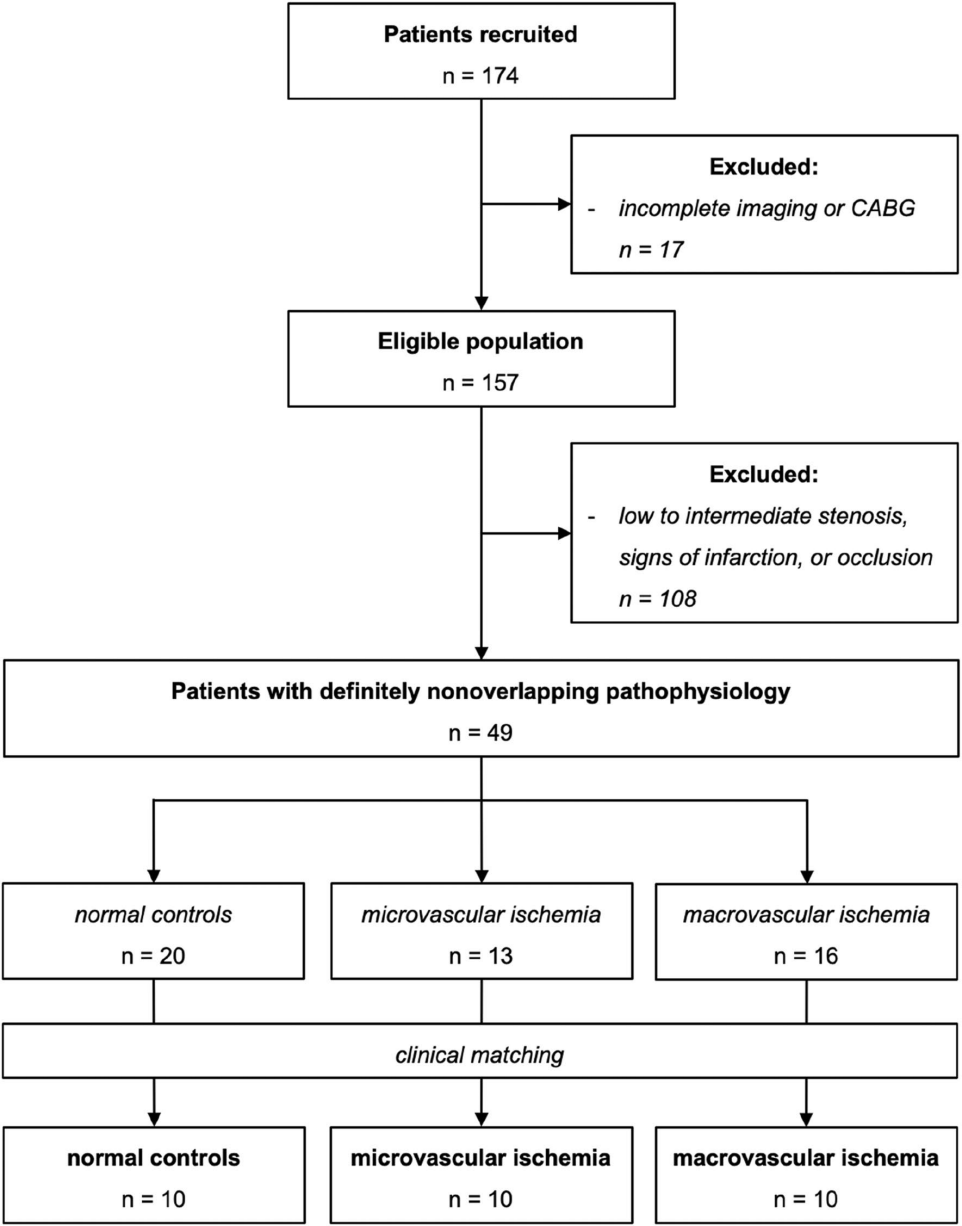
Flow chart of patient selection to assemble a rigorously defined cohort with nonoverlapping perfusion pathophysiology. Remaining eligible cases were selected by clinical matching, if possible.

We used a combined reference standard consisting of invasive catheterization with quantitative coronary angiography (QCA) and fractional flow reserve (FFR) measurement in conjunction with focal perfusion defects on 4D-CTP assessed visually. Each visual perfusion defect on 4D-CTP was correlated with invasive results. Perfusion defects have been assessed by two readers in a consensus procedure and have been validated by an independent third reader. We defined the following criteria to assign patients to either of the three perfusion groups (see also flow chart in Fig. 1): Macrovascular ischemia was defined as visual ≥ 90% diameter stenosis on angiography or ≥ 50% diameter stenosis with positive fractional flow reserve (FFR) (< 0.8) in conjunction with visually abnormal perfusion in the downstream myocardium on 4D-CTP. For this study, we excluded patients with occlusive disease. Microvascular ischemia was defined as < 25% diameter stenosis despite visual subendocardial perfusion abnormality on plain 4D-CTP. With this approach we expect to identify patients with subendocardial ischemia due to microvascular atherosclerosis, which has been found to be the most common pathophysiological entity in patients with microvascular ischemia^2^. This subset can be described with the term “ischemia and nonobstructive coronary arteries” (INOCA)^22^. Patients with 25–49% diameter stenosis were excluded to avoid potential overlap of pathophysiology. Normal controls were defined as normal coronary arteries without signs of perfusion deficits on 4D-CTP. Segments with signs of delayed enhancement were excluded from analysis.

### Comparison of fractal analysis to myocardial blood flow

To assess CAD detection, we compared fractal analysis to CT-derived myocardial blood flow (MBF) for identifying hemodynamically relevant stenosis using invasive angiography and FFR as reference standard. Analysis was conducted on a per-segment level using the American Heart Association (AHA) 17-segments model. Three nonoverlapping patient groups – one with normal perfusion, one with macrovascular ischemia, and one with microvascular ischemia – were retrospectively extracted from the dataset to establish reference values for differentiation of normal and abnormal perfusion states using fractal analysis.

### Imaging protocol

The imaging procedures included 4D-CTP, coronary CT angiography (CCTA), delayed enhancement CT (CTDE), and invasive coronary angiography with FFR measurement as well as cardiac magnetic resonance imaging (MRI) with a dynamic contrast-enhanced perfusion sequence in a subset of patients. All participants gave written informed consent. The study was approved by an institutional review board (Clinical Research Ethics Review Committee of Mie University Hospital). FM, MD and KK had full access to all data in the study and take responsibility for data integrity and analysis.

Cardiac CT examinations were performed on a second- or third-generation dual-source CT scanner (Somatom Definition Flash, Somatom Force, Siemens Healthineers, Germany). Patients were required to be fasting for at least 4 h and abstain from caffeine at least 12 h before the CT examination. The 30-s 4D-CTP scan was acquired using the ECG-triggered shuttle mode and started with the injection of 40 mL of contrast medium with an iodine concentration of 370 mg/mL at a flow rate of 5 mL/s, followed by a 40 mL saline flush during the administration of 20 mg of adenosine triphosphate (ATP) at 160 μg/kg/min for > 3 min. Dynamic 4D-CTP in the second- or third-generation CT scanner was performed with the following acquisition parameters: collimation = 32 × 1.2 or 48 × 1.2 mm, rotation time = 0.28 or 0.25 s, and tube voltage = 80 or 70 kV, respectively. Tube current was determined using an automatic exposure control system with a quality reference of 350 mAs/rotation at 120 kV.

In this study, the 4D-CTP datasets were assessed by fractal analysis (FA) as described in detail below, and invasive coronary angiography with FFR and visual analysis of 4D-CTP served as the combined reference standard.

### Image preprocessing

Image preprocessing was performed in several steps in order to adequately assess four-dimensional, dynamic contrast-enhanced CT data reconstructed on a 512 × 512 matrix with around 70 slices at 12–15 time points. The steps included (I) registration for motion correction with dynamic contrast enhancement as a major challenge, (II) image denoising with individual adaptation to image quality while retaining of precious local signal variation, (III) image standardization to ensure intersubject comparability, and (IV) segmentation to constrain fractal analysis to the myocardium. (I) For image registration, the open-source library SimpleElastix was employed (Marstal K. 2015. https://simpleelastix.github.io/). A principal-component analysis metric (PCAMetric2) was used to account for contrast changes over time. This approach explicitly accounts for temporal signal variability due to contrast agent administration and ensures that anatomical features are retained despite contrast uptake and washout. (II) Image denoising comprised a locally constrained median filter with a radius of 1 (i.e., a 3 × 3 pixel kernel) in combination with bilateral filtering adapted to individual noise levels. For bilateral filtering, spatial standard deviation was set to σ_domain_ = 2 and intensity standard deviation σ_range_ was determined from the standard deviation of the unenhanced myocardium to account for individual image quality^23^. (III) Image standardization was performed by converting CT Hounsfield units into absolute contrast agent concentration^24^. (IV) Segmentation of the left ventricular myocardium was performed semiautomatically by sparsely annotating the myocardium in short-axis multiplanar reformation, and linear contour interpolation was used to automatically connect the sparse segmentations. Myocardial segmentation at this stage, however, only limited the area, which was subjected to fractal analysis in order to speed up calculation. Actual quantitative myocardial measurements were performed after fractal analysis as explained below. The resulting preprocessed dataset was subsequently subjected to fractal analysis.

### Fractal analysis

Fractal analysis was performed in a local manner based on the approach in^25^. This study extends said approach by additionally considering the third, spatial dimension as well as the fourth, temporal dimension, therefore, our method constitutes four-dimensional (4D) fractal analysis. The algorithm can be described as follows, starting with the two-dimensional case: a single image slice can be considered a texture embedded in two-dimensional space with intensity as the third dimension. Fractal analysis evaluates feature propagation over multiple scales, which can be represented by two blankets molded to the texture. One blanket is iteratively raised, the other lowered from the texture, losing detail in the process. The local fractal dimension is obtained from quantifying the loss of detail (represented by the surface area of the blankets) as a function of distance to the original texture^25,26^. For 4D fractal analysis, the image can be considered a hypertexture embedded in four-dimensional space with intensity constituting a fifth dimension. Molding the two hyperblankets works analogously to the two-dimensional case with loss of detail occurring in all neighboring dimensions according to the formulas:

1 $${u}_{\varepsilon }\left(i,j,k,t\right)=\mathit{max}\left\{{u}_{\varepsilon -1}\left(i,j,k,t\right)+1,\underset{\left|\left(m,n,o,s\right)-\left(i,j,k,t\right)\right|\le 1}{\mathit{max}}{u}_{\varepsilon -1}\left(m,n,o,s\right)\right\}$$

2 $${b}_{\varepsilon }\left(i,j,k,t\right)=\mathit{min}\left\{{b}_{\varepsilon -1}\left(i,j,k,t\right)-1,\underset{\left|\left(m,n,o,s\right)-\left(i,j,k,t\right)\right|\le 1}{\mathit{min}}{b}_{\varepsilon -1}\left(m,n,o,s\right)\right\}$$

where u_ε_ and b_ε_ represent the top and bottom blankets, respectively; ε the scale, i.e., the counter of iterations; and i, j, k, t as well as m, n, o, s are pixel coordinates (in 3D plus time). Unlike the area in the two-dimensional case, the hypervolume V(ε) of the blankets is calculated at each iteration:

3 $$V\left(\varepsilon \right)=\frac{{\sum }_{i,j,k,t}\left({u}_{\varepsilon }\left(i,j,k,t\right)-{b}_{\varepsilon }\left(i,j,k,t\right)\right)}{2\varepsilon }$$

Repeating this process yields a bi-logarithmic linear relationship with a decreasing slope for ln V(ε) against ln ε. The slope is determined from a linear fit of log V(ε) against log ε and FD can be calculated as:

4 $$FD=4-slope$$

During the process, fractal analysis integrates and thereby collapses the temporal dimension, which results in a spatial FD map reflecting complexity of the perfusion pattern in local vicinity over time.

A per-segment analysis of FD was performed by selecting a representative region of interest (ROI) from each myocardial segment based on the AHA-17-segments model. Mean FD of each segment was subjected to statistical analysis. Two readers with over 15 years or, respectively, over 6 years experience in cardiovascular imaging independently performed fractal analysis to assess inter-reader variability. Both readers were blinded to any clinical information including perfusion status.

### Perfusion rate estimation

A local perfusion map was computed based on a surrogate of myocardial blood flow from adenosine-induced maximum hyperemia in dynamic CT perfusion scans using the maximum upslope method (MUS)^27–30^. The CT scan was preprocessed as described above, except that a radius of 3 was used for median filtering. The MUS was used with a surrogate for myocardial blood flow being estimated from the ratio of the myocardial to arterial upslope during the first-pass contrast agent uptake phase:

Perfusion_MUS_ = max(Ṡ_myo_)/max(S_AIF_) (5).

where S denotes the time-to-signal intensity curve, Ṡ is its first derivate with the maximum being obtained from a linear curve fit of the upslope, and AIF denotes the arterial input function measured in the aorta. The same ROIs as for fractal analysis were used for quantitative MBF measurements.

### Statistical analysis

Three patient-wise pathophysiological groups were defined (i.e., normal perfusion, macrovascular ischemia, and microvascular ischemia). According to the pathophysiological hypothesis, a correlation between FD and the underlying perfusion pattern was postulated. The Kruskal–Wallis test and pairwise group comparisons using the post hoc Mann–Whitney U-test were performed to test for group-wise differences. Intrapatient clustering due to analysis on a per-segment level was eliminated by averaging. To derive optimal cutoffs for the individual pathophysiological groups, the optimal point was calculated as part of a multiclass receiver operating characteristic (ROC) analysis after elimination of intrapatient clustering. Myocardial perfusion was correlated with both the pathophysiological groups and FD. Agreement with pathophysiology was evaluated by calculating quadratic-weighted κ^31^. Inter-reader variability of fractal analysis was assessed using Cohen’s κ for two readers. Moreover, a Bland–Altman analysis was carried out. For diagnostic accuracy, sensitivity and specificity were calculated and compared between fractal analysis and MBF using the McNemar test. Multi-class area under the receiver operating curve (AUC) was calculated by considering normal perfusion, microvascular CMD, and macrovascular CAD. A level of p ≤ 0.05 was considered significant, and adjusted p-values with Bonferroni correction, where appropriate, are reported. The STARD guidelines were adhered to. Statistical analysis was performed with R (v3.4.1, R Foundation for Statistical Computing. Vienna, Austria).

## Results

### Patient cohort

The characteristics of the patient cohort are summarized in Table 1. Hemodynamic response characteristics during 4D-CTP can be found in Table 2. Each of the 17 myocardial segments per patient were classified by pathophysiology: In normal controls, 170 segments were included. In patients with microvascular disease, 123 ischemic segments were identified and allocated to the microvascular ischemia group. In patients with macrovascular CAD, 93 ischemic segments, or, respectively, 15 vessels with significant CAD according to above criteria were identified and allocated to the macrovascular CAD group.

**Table 1:** Patient characteristics. SD—standard deviation, PCI—percutaneous coronary intervention.

Characteristic	Normal (n = 10)	Microvascular (n = 10)	Macrovascular (n = 10)
Male	4	8	6
Age (mean ± SD)	68.7 ± 8.4	66.7 ± 7.8	69.6 ± 12.0
Body mass index (mean ± SD)	22.8 ± 3.5	23.7 ± 3.8	24.0 ± 3.3
**Coronary risk factors**			
Hypertension	4	7	10
Dyslipidemia	4	5	8
Diabetes mellitus	2	0	8
Smoking	4	7	6
Family history of CAD	2	3	1
**Symptoms**			
Typical angina	3	3	3
Atypical angina	6	1	0
Non-anginal pain	0	3	0
Dyspnea	1	0	1
History of PCI	0	1	4
History of myocardial infarction	0	0	2

**Table 2:** Hemodynamic response to ATP.

Parameter	Normal (n = 10)	Microvascular (n = 10)	Macrovascular (n = 10)
**During stress**			
Systolic blood pressure (mmHg)	131.1 ± 12.3	119.0 ± 27.1	120.5 ± 13.9
Diastolic blood pressure (mmHg)	73.3 ± 11.6	59.4 ± 15.4	63.6 ± 9.4
Heart rate (beats/min)	76.6 ± 17.3	80.1 ± 13.3	90.4 ± 33.0
**At rest**			
Systolic blood pressure (mmHg)	138.5 ± 19.0	139.9 ± 24.1	139.1 ± 18.7
Diastolic blood pressure (mmHg)	76.6 ± 9.0	72.9 ± 12.3	71.2 ± 12.9
Heart rate (beats/min)	64.2 ± 11.7	64.3 ± 7.1	73.5 ± 13.6

**Figure 2:**
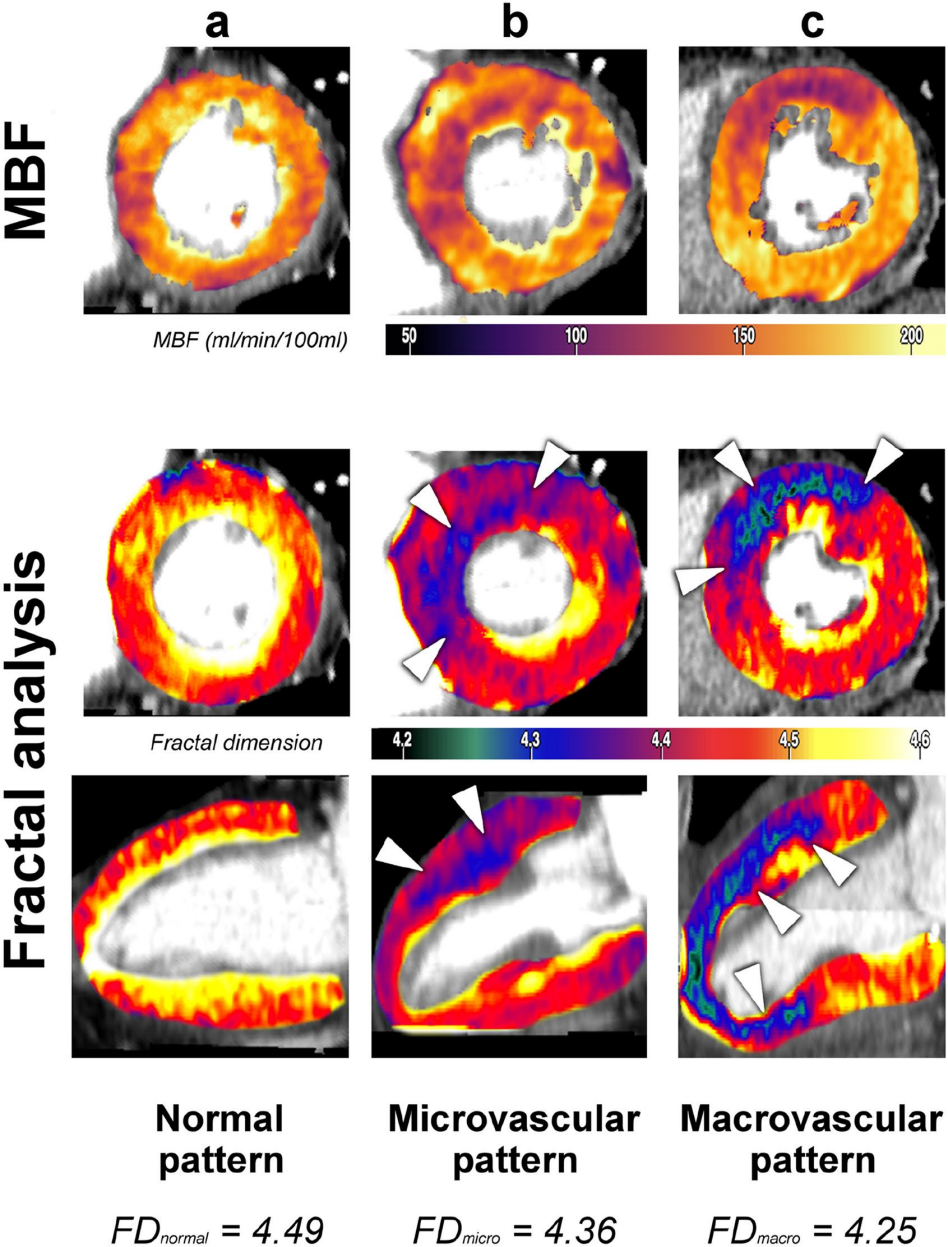
Comparison of myocardial blood flow estimation (first row) and fractal analysis (second and third row) of left-ventricular myocardium in three patients with either normal perfusion (**A**), microvascular ischemia (**B**) or macrovascular coronary artery disease (CAD, C). (**A**) Normal perfusion pattern with physiological FD (perfusion complexity) and MBF = 146 ml/min/100 ml (female, age 69, hypertension, dyslipidemia, smoking, family history of CAD). (**B**) Microvascular ischemia pattern in the anterior wall and septum with normal coronary arteries and MBF = 100 ml/min/100 ml in the ischemic area (female, age 69, hypertension, diabetes mellitus, smoking). (**C**) Macrovascular ischemia pattern in the territory of the left anterior descending artery (LAD) and a corresponding 90% stenosis (segment 6, FFR < 0.8) on invasive coronary angiography and MBF = 94 ml/min/100 ml in the ischemic area (female, age 75, hypertension, dyslipidemia, diabetes mellitus, smoking). MBF identified ischemic regions, however, only FD correctly differentiated between microvascular and macrovascular perfusion patterns.

The complete analysis procedure took about 20 min per patient, which included registration, denoising, myocardial segmentation, calculation of FD and MBF maps, and definition of myocardial regions of interest according to the AHA 17-segments model. Representative analysis results for patients from each pathophysiological group are depicted in Fig. 2.

### Fractal analysis

**Figure 3:**
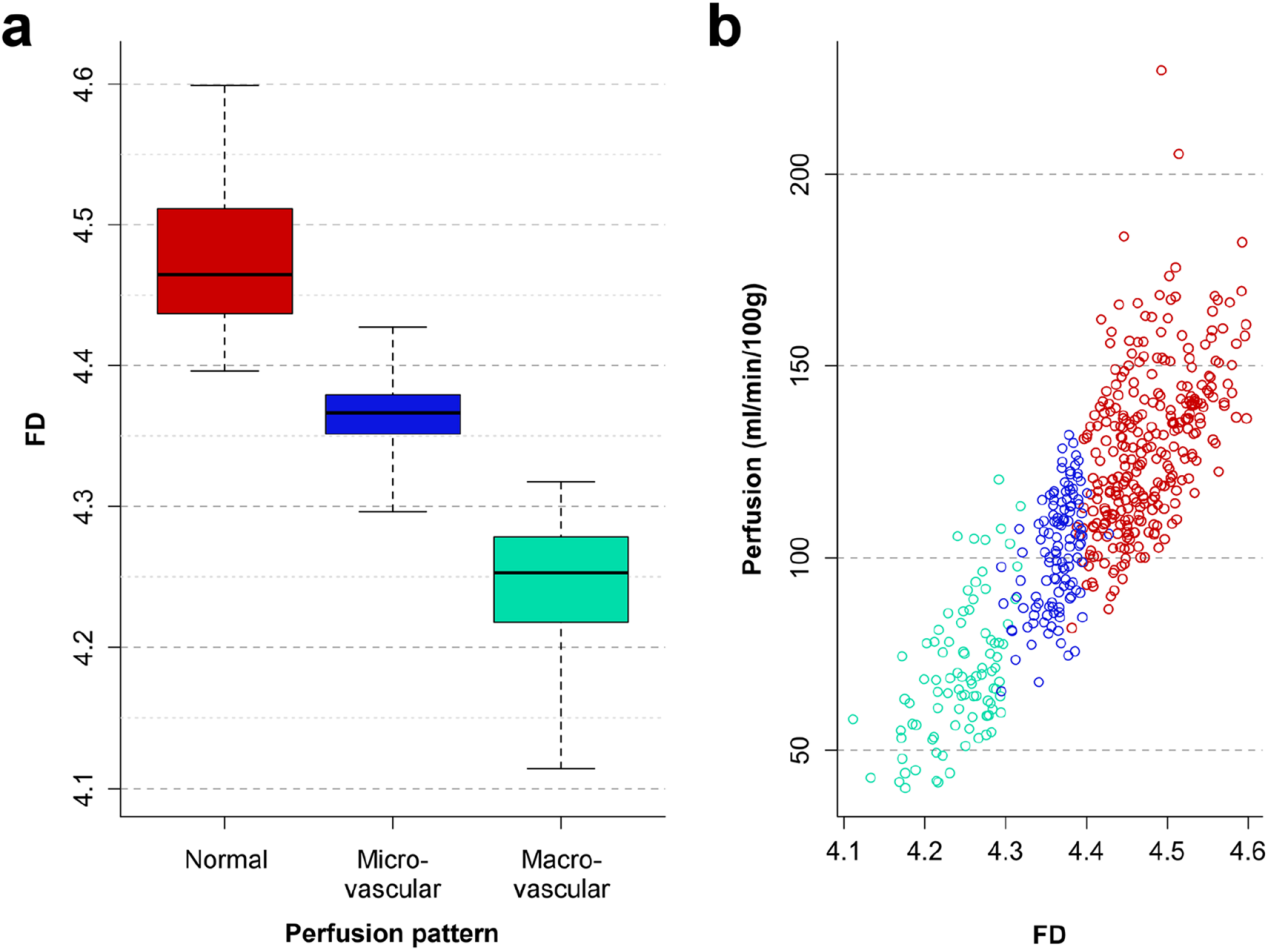
Boxplot of fractal dimension (FD) versus perfusion pattern (**A**) and myocardial perfusion (semiquantitative maximum upslope estimate (**B**). (**A**) Fractal dimension was significantly (p < 0.001) different between normal perfusion, microvascular and macrovascular ischemia patterns (thresholds see Table 3 and Results section). (**B**) Fractal dimension showed moderate linear correlation with perfusion, however with a relatively high variation in perfusion.

In patients without ischemia, perfusion complexity was high (FD_normal_ = 4.49, interquartile range, IQR_normal_: 4.46–4.53), whereas perfusion complexity was significantly reduced in ischemia (p < 0.001, Fig. 3A): We found a moderate reduction in microvascular ischemia (FD_micro_ = 4.37, IQR_micro_: 4.36–4.37) and a strong reduction in macrovascular CAD (FD_macro_ = 4.26, IQR_macro_: 4.24–4.27) with a squared multiple regression coefficient r^2^ = 0.81. FD predictions agreed excellently with pathophysiology (κ = 0.96, 95%-confidence interval [CI]: 0.94–0.97). The optimal cutoff threshold to differentiate normal perfusion and ischemia (i.e., pooled micro- and macrovascular) was FD = 4.41, and for differentiating micro- and macrovascular ischemia FD = 4.31, respectively (Table 3).

**Table 3:**
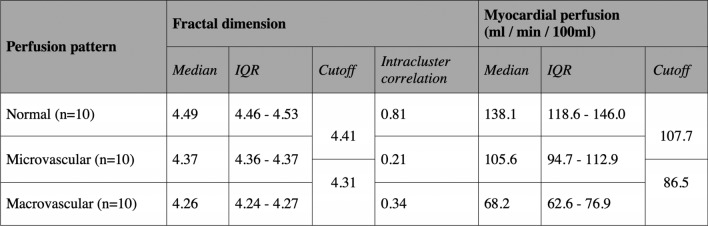
Results of fractal analysis for detecting and classifying ischemia. The fractal dimension (FD) is the quantitative parameter for complexity of perfusion and is given after elimination of intra-patient clustering. IQR—interquartile range.

### Inter-reader variability of fractal analysis

**Figure 4:**
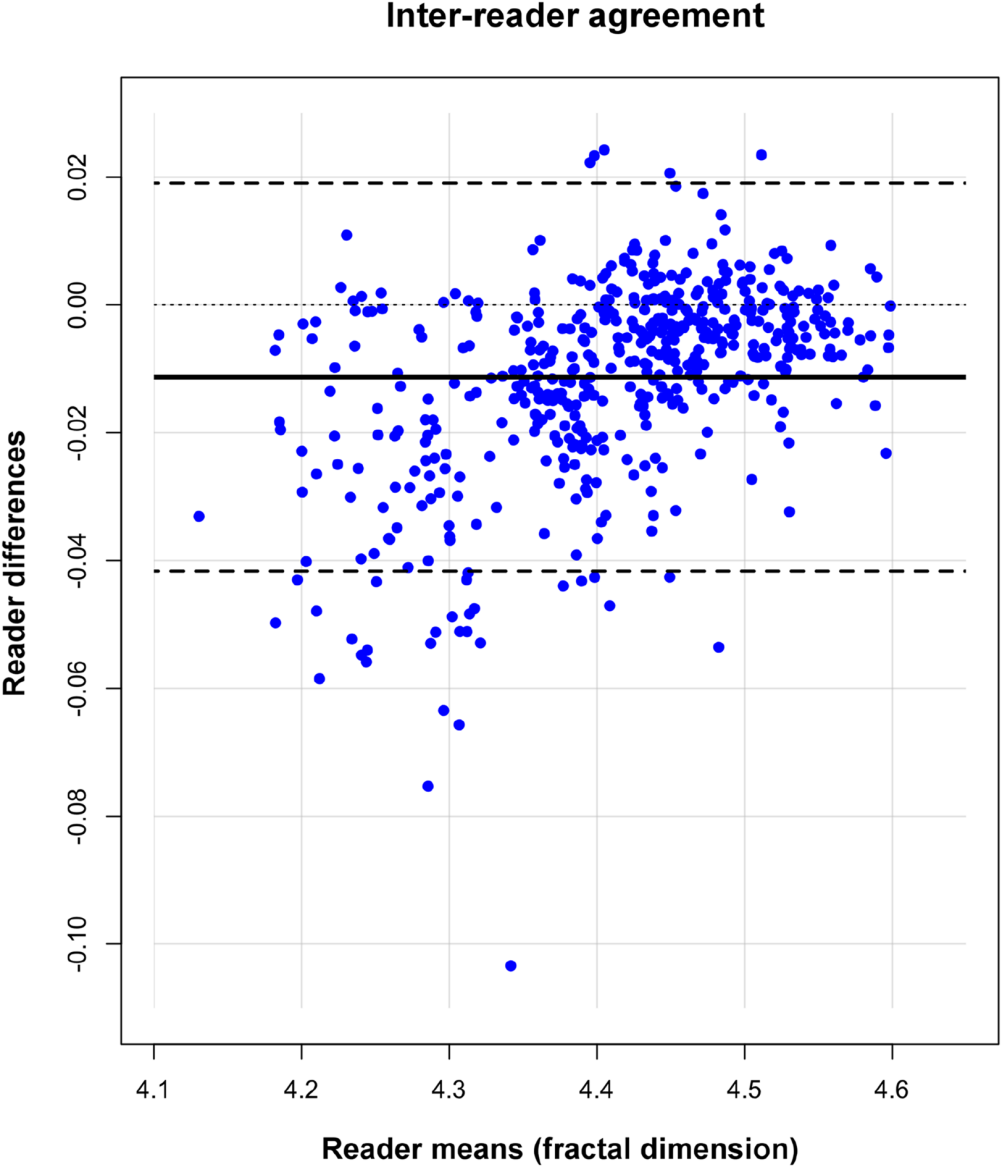
Bland–Altman plot for inter-reader agreement of fractal analysis per myocardial segment. Two readers (> 15 years and > 5 years of experience in cardiovascular imaging) independently performed fractal analysis while blinded to any clinical information and perfusion status. The more experienced reader is considered as reference.

The fractal analysis results from two independent readers agreed excellently (Cohen’s κ = 0.94, CI: 0.92–0.96) without relevant bias (−0.011, CI: −0.012 to −0.010) and acceptable limits of agreement (−0.04 to 0.02) with the more experienced reader being considered as reference (Fig. 4).

### MBF and relation to fractal analysis

The MBF estimate was significantly different between the three pathophysiological perfusion states (p < 0.001) with the median MBF being lower in ischemia (MBF_micro_ = 105.6 ml/min/100 ml, IQR: 94.7–112.9 versus MBF_macro_ = 68.2 ml/min/100 ml, IQR: 62.6–76.9) compared with normal perfusion status (MBF_normal_ = 138.1 ml/min/100 ml, IQR: 118.6–146.0), see Table 3. Comparison of MBF and FD yielded a moderate linear correlation (r^2^ = 0.71, see Fig. 3B) with a tendency of perfusion to become less chaotic at reduced MBF levels. MBF agreed moderately with pathophysiology (κ = 0.77, 95%-confidence interval, [CI]: 0.72–0.81). The optimal cutoff threshold to differentiate normal perfusion and ischemia (i.e., pooled micro- and macrovascular) was MBF = 107.7 ml/min/100 ml, and for differentiating micro- and macrovascular ischemia MBF = 86.5 ml/min/100 ml, respectively.

### Detection of coronary artery disease

For the detection of CAD, we dichotomized the dataset into macrovascular CAD (n = 10) and non-CAD (n = 20, i.e., pooled non-ischemia and microvascular ischemia). We assigned a patient or, respectively, a vessel to CAD when FD in at least one pertaining myocardial segment was below the FD threshold for CAD (i.e., FD ≤ 4.31), yielding 15 CAD vessels and 75 non-CAD vessels.

On the patient level, we found that fractal analysis correctly identified 10/10 CAD patients and 17/20 non-CAD patients, corresponding to a sensitivity of 100% (CI: 69–100%) and a specificity of 85% (CI:62–97%). In comparison, MBF correctly identified 10/10 CAD patients and 5/20 non-CAD patients, corresponding to a sensitivity of 100% (CI: 69–100%) and a specificity of 25% (CI: 9–49%).

On the vessel level, fractal analysis correctly classified 15/15 CAD vessels and 71/75 non-CAD vessels, yielding a sensitivity of 100% (CI: 69–100%) and a specificity of 95% (CI:87–99%). MBF correctly classified 15/15 CAD vessels and 24/75 non-CAD vessels, corresponding to a sensitivity of 100% (CI: 78–100%) and a specificity of 32% (CI: 22–44%).

While both fractal analysis and MBF perfectly diagnosed CAD in this highly selective patient cohort, fractal analysis significantly outperformed MBF in terms of specificity for identifying non-CAD patients (p = 0.02) and non-CAD vessels (p < 0.001).

### Differentiation of coronary artery disease and microvascular ischemia

For the differentiation of macrovascular CAD and microvascular CMD, we analyzed the respective sub-cohorts (each n = 10). For diagnostic accuracy, we defined macrovascular CAD as positive outcome. Fractal analysis correctly differentiated CAD and CMD in the majority of myocardial segments, i.e., correct classifications were achieved in 93/93 CAD segments and 113/123 CMD segments, yielding a sensitivity of 100% (CI: 96–100%), a specificity of 92% (CI: 86–96%) and Cohen’s κ = 0.91 (CI: 0.85–0.96).

In comparison, MBF correctly classified 79/93 CAD segments and 102/123 CMD segments, yielding a sensitivity of 85% (CI: 76–92%), a specificity of 83% (CI: 75–89%) and Cohen’s κ = 0.69 (CI: 0.59–0.79).

The multi-class AUC (normal vs. microvascular vs. macrovascular ischemia) on the myocardial segment level was 0.98 (CI: 0.97–0.99) for fractal analysis and 0.78 (CI: 0.76–0.81) for MBF.

## Discussion

This proof-of-concept study introduces fractal analysis of 4D-CTP for differentiating microvascular and macrovascular causes of myocardial ischemia in a rigorously characterized patient cohort. Our concept is strictly based on pathophysiological considerations, which have been well studied in normal perfusion and animal models, however, those findings have not been put to clinical use before. In comparison to MBF estimation, fractal analysis allows to reliably identify microvascular ischemia and, moreover, improves the diagnostic accuracy for detecting macrovascular CAD. As quantified by FD, perfusion is inherently complex with complexity being reduced in ischemia, which depends on the underlying pathophysiology. Perfusion complexity is only partially related to MBF and provides independent information on the perfusion status. Reference values for fractal dimension (FD) have been established in this study and are readily applicable to clinical pharmacological stress 4D-CTP protocols to complement noninvasive imaging workup in patients with stable myocardial ischemia.

The differentiation of macrovascular and microvascular ischemia is thought to reflect important prognostic implications: stable myocardial ischemia patients with a microvascular component have a poorer clinical outcome and prognosis including a higher rate of major adverse clinical events, which is specifically evident in female patients^32,33^, and is an area of active research^34^. Various diagnostic methods have been proposed to identify microvascular ischemia by exploiting its different pathophysiological mechanisms. However, no single noninvasive method has yet reached clinical applicability in terms of enabling a reliable diagnosis of microvascular ischemia. In current clinical practice, the diagnosis of microvascular ischemia is based on the presence of myocardial ischemia on functional perfusion imaging, i.e., MRI^35–37^, CTP^38^, or positron emission tomography (PET)^39^, in the absence of coronary stenosis on either coronary angiography including an option for acetylcholine testing^40^ or CCTA, which has high diagnostic accuracy for noninvasive detection of macrovascular CAD^41,42^. While the pathophysiological relation between epicardial vessel wall remodeling and resultant pathophysiological consequences for myocardial blood flow (MBF) has been studied^43^, imaging of the microcirculation remains challenging. Since CAD is often diffuse with associated mild to moderate stenoses, the distinction between ischemia primarily due to macrovascular stenosis or microvascular ischemia is difficult. As demonstrated in the current study, fractal analysis of 4D-CTP may provide a quantitative imaging parameter that allows differentiation of macrovascular and microvascular ischemia, thereby contributing to the demand for a more personalized, non-invasive disease characterization for individualized therapeutic interventions^44^.

Fractal analysis of perfusion has shown potential to differentiate the predominant pathophysiology underlying myocardial ischemia^19^. The principle assumes that structural and functional alterations of the vasculature alter the perfusion pattern, which in turn can be assessed using radiological and nuclear medicine imaging methods^18^. The vascular scale that is impaired determines how the perfusion pattern is altered. In macrovascular ischemia, stenoses in the large epicardial arteries restrict blood flow to the downstream perfusion territory. The net effect on flow exhausts vasodilative capacity due to a global reduction of driving pressure for the diseased perfusion territory. In contrast, microvascular ischemia is the result of heterogeneous endothelial dysfunction and impaired microvascular function with diffuse atherosclerosis being considered the most common cause^2,5^. Moreover, microvascular ischemia is characterized by a patchy distribution pattern throughout the myocardium with healthy and diseased vascular beds existing side by side^21,45–48^. Those characteristics result in a patchy exhaustion of vasodilative capacity with potential for compensation by unaffected neighboring microvascular beds. The involvement of different vascular scales in macrovascular (i.e., large epicardial arteries) and microvascular (i.e., small arteries and arterioles) ischemia alters the perfusion pattern in different ways and is observable in terms of contrast agent deposition. Fractal analysis is a promising method to quantitatively assess this alteration of the perfusion pattern, given the fractal nature of both the structural vascular tree^49^ and perfusion as its functional correlate^16,50^. Indeed, we found a significant difference in fractal dimension between macrovascular and microvascular ischemia.

While the perfusion rate is a good marker of the severity of ischemia, it does not reflect the vascular scale that is impaired. Therefore, MBF did not provide reliable differentiation of the underlying pathophysiology in our study. For example, a perfusion rate of 100 ml/min/100 ml was observed in all three pathophysiological groups we analyzed, as shown in Fig. 3B. Moreover, different methods for perfusion modelling exist, and they vary widely in terms of validity and quantitative results^51^. Therefore, it would be difficult to establish a single MBF threshold for diagnosis of the underlying pathophysiology. Unlike MBF estimation, fractal analysis does not depend on comparison to individual physiological MBF levels or hemodynamic model fitting. Therefore, the threshold we established for FD in this study might be less susceptible to interindividual differences and is therefore straightforward to apply to dynamic 4D-CTP scans. Fractal analysis complements MBF analysis and contributes to the quantification of changes in perfusion patterns reflecting the underlying pathophysiology. Moreover, fractal analysis has potential to improve especially specificity for detecting coronary artery disease compared to MBF as suggested by our data.

Our retrospective study design and rigorous inclusion criteria aim at a pathophysiologically nonoverlapping patient selection to investigate different perfusion conditions in their isolated form. However, this approach entails several limitations: First, the number of included patients per group is small. However, the sample has been taken from the large, prospective AMPLIFiED multi-center study and strict selection criteria were necessary to ensure a consistent definition of perfusion pathophysiology. In turn, our patient selection process introduced a selection bias with regards to uniformity of underlying pathophysiology and neglects intermediate stenosis grades. Moreover, our design does not allow to draw conclusions on prospective patient outcome, and it does not reflect the majority of patients encountered in clinical routine. A retrospective study with less rigorous inclusion criteria, as well as a prospective study would be reasonable to assess the value of fractal analysis in a less uniform patient cohort, and, respectively, to explore potential prognostic implications of fractal analysis. Moreover, the number of patients is relatively small with only 10 patients in each of the three pathophysiological groups. However, patients with isolated microvascular ischemia and normal coronary arteries are relatively uncommon^22,52^. The analysis of the myocardial segment level introduces intrapatient clustering, which we mitigated by evaluating the amount of clustering and adequately adjusting for it.

15O-water PET is usually regarded as a gold standard for myocardial perfusion estimation. However, since PET was not available from the investigated study cohort, the calculated MBF values could not be validated against a reference. However, previous studies have established correlations of perfusion rate estimates from 4D-CTP and PET, e.g., in^53^.

Fractal analysis as presented in this paper identifies the vascular scale which is responsible for perfusion impairment. Further research would be desirable to investigate whether fractal analysis could help to quantify the individual contribution of macro- and microvascular components in complex cases, which might assist in clinical decision making. Moreover, prospective testing of such a stratification approach would be required to explore the impact on prognosis and outcome and to establish a high level of evidence for fractal analysis of perfusion^54^.

## Conclusion

Perfusion is inherently complex under physiological conditions and is less so in patients with ischemia. Fractal analysis allows to quantify perfusion complexity, thus enabling to differentiate micro- and macrovascular causes of ischemia. In relation to MBF estimation, fractal analysis further improves the detection of significant macrovascular CAD. The results of our study might be exploited in the noninvasive imaging workup of patients with chronic myocardial ischemia and thus help in guiding clinical management and stratifying patients by pathophysiology. It remains to be shown by further research whether fractal analysis of perfusion also has a prognostic benefit and potential to improve clinical outcome.

## Data Availability

The datasets generated during and/or analyzed during the current study are available from the corresponding author on reasonable request.
